# Clinical significance of transmembrane 4 superfamily in colon cancer

**DOI:** 10.1038/sj.bjc.6601015

**Published:** 2003-07-01

**Authors:** H Hashida, A Takabayashi, T Tokuhara, N Hattori, T Taki, H Hasegawa, S Satoh, N Kobayashi, Y Yamaoka, M Miyake

**Affiliations:** 1 Department V of Oncology and Department of Thoracic Surgery, Kitano Hospital, Tazuke Kofukai Medical Research Institute, 2-4-20, Ohgimachi, Kita-ku, Osaka 530-8480, Japan; 2 Department of Surgery, Kitano Hospital, Tazuke Kofukai Medical Research Institute, 2-4-20, Ohgimachi, Kita-ku, Osaka, 530-8480, Japan; 3 First Department of Internal Medicine, Ehime University School of Medicine, Oazashizukawa, Shigenobu-cho, Onsen-gun, Ehime 791-0295, Japan; 4 First Department of Surgery, Ehime University School of Medicine, Oazashizukawa, Shigenobu-cho, Onsen-gun, Ehime 791-0295, Japan; 5 Department of Gastroenterological Surgery, Kyoto University Graduate School of Medicine, 54, Shogoin Kawahara-cho, Sakyo-ku, Kyoto 606-8507, Japan

**Keywords:** TM4SF, MRP-1/CD9, KAI1/CD82, CD151, colon cancer

## Abstract

Cell motility is an important cellular function closely related to the processes of tumour progression and metastasis. Several members of transmembrane 4 superfamily (TM4SF) have been reported to be associated with cell motility and metastatic potential of solid tumour. The aim of this study is to clarify the clinical significance of the member of TM4SF (MRP-1/CD9, KAI1/CD82 and CD151) in human colon cancer. We studied 146 colon cancer patients who underwent curative surgery and studied the expression of MRP-1/CD9, KAI1/CD82 and CD151 using reverse transcriptase – polymerase chain reaction and immunohistochemistry. We found that 64 patients (43.8%) had *MRP-1/CD9*-positive tumours and that the overall survival rate of patients with *MRP-1/CD9*-positive tumours was much higher than that of patients with *MRP-1/CD9*-negative tumours (89.8 *vs* 50.8%, *P<*0.001). In contrast, 63 patients (43.2%) had *KAI1/CD82*-positive tumours and the overall survival rate of patients with *KAI1/CD82*-positive tumours was also higher than that of patients with *KAI1/CD82*-negative tumours (84.8 *vs* 54.9%, *P*=0.002). On the other hand, positive *CD151* expression had a bad effect on the overall survival rate of patients with colon cancer (61.2 *vs* 74.9%, *P*=0.022). In a multivariate analysis, *MRP-1/CD9* status was a good indicator of the overall survival (*P*=0.007). We have shown that the reduction of MRP-1/CD9 and KAI1/CD82 expression, and the increasing CD151 expression are indicators for a poor prognosis in patients with colon cancer. This is a first report describing about the relation between CD151 and colon cancer.

Cell motility plays an important key function related to the process of tumour progression and metastasis (Miyake and Hakomori, 1991). It is partially dependent on adhesion molecules and proteases (Hashida *et al*, 2001,2002a). Previously, we reported that the motility-related protein-1 (MRP-1) is an antigen recognised by monoclonal antibody (MAb) M31-15 which inhibits cell motility and the MRP-1 sequence coincides with the cluster of differentiation antigen 9 (CD9) (Miyake *et al*, 1991). In our previous reports, we showed MRP-1/CD9-overexpressing tumour cells negative cell motility and metastatic potential (Ikeyama *et al*, 1993). Therefore, MRP-1/CD9 regulates cell motility and is a receptor for negative signal ligands. In addition, negative MRP-1/CD9 expression was associated with a poor prognosis in breast cancer (Miyake *et al*, 1996), lung cancer (Higashiyama *et al*, 1995) and pancreatic cancer (Sho *et al*, 1998). These data also suggest that MRP-1/CD9 expression might be associated with metastatic ability and degree of malignancy. MRP-1/CD9 belongs to the transmembrane 4 superfamily (TM4SF), which is characterised by four transmembrane domains delimiting two extracellular regions of unequal size, as well as a particular fold in the large extracellular loop (Wright and Tomlinson, 1994).

KAI1/CD82 is also a member of TM4SF. KAI1/CD82 expression suppressed experimental metastasis of rat prostate tumour cells (Dong *et al*, 1995), and decreased motility and invasion of colon carcinoma cells (Takaoka *et al*, 1998). KAI1/CD82 is considered to be a metastasis-suppressor gene of prostate cancer and low *KAI1/CD82* expression has been reported to be involved in the malignant progression of prostate cancer (Dong *et al*, 1996). We also showed that decreased *KAI1/CD82* gene expression was an indicator of poor prognosis in lung cancer (Adachi *et al*, 1996), breast cancer (Huang *et al*, 1998) and pancreatic cancer (Sho *et al*, 1998). These data show that KAI1/CD82 is an important tumour suppressor gene in cancer metastasis and progression.

On the other hand, CD151 is also a transmembrane molecule that has been characterised as a member of the evolutionally conserved TM4SF, and it is known as SFA-1 and PETA-3 (Fitter *et al*, 1995; Hasegawa *et al*, 1996). CD151 cDNA shows an open reading frame of 253 amino acids that encodes a protein of molecular mass 28 kDa. In addition, human CD151 gene locates on chromosome 11p15.1. CD151 in involved in cell adhesion, cell motility, metastasis, and stability and formation of hemidesmosomes (Yauch *et al*, 1998).

Several members of TM4SF are associated with the metastatic phenotype. In addition, for the most part, they work negatively. Recently, it was reported to be the first member of the TM4SF to show signs of being a positive effecter of metastasis (Testa *et al*, 1999). Moreover, CD151 enhances cell motility and cancer metastasis (Kohno *et al*, 2002) and CD151 overexpression leads to a poor prognosis of the patients with non-small cell lung cancer (Tokuhara *et al*, 2001). Therefore, CD151 is a metastasis-associated antigen that appears to contribute to the metastatic phenotype positively. These findings set *CD151* apart from *MRP-1/CD9* and *KAI1/CD82* that appear to act as metastasis-suppressor genes. It may contribute to the collapse of tetraspanin/tetraspanin complexes. In addition, no consistent findings have been reported as a prognostic indicator for *CD151* gene in colon cancer. As part of our evaluation of members of the TM4SF as possible prognostic predictors, we performed a retrospective study on the expression of the *MRP-1/CD9* gene, the recently identified *KAI1/CD82* gene and the *CD151* gene in human colon cancer.

## MATERIALS AND METHODS

### Clinical characteristics of the patients

We studied 146 patients with up to stage III colon cancer who had undergone surgery at the Department of Surgery of the Kitano Hospital between October 1994 and May 2001. All patients underwent curative surgery. The postsurgical staging of each tumour was classified according to the tumour–node–metastasis (TNM) staging system (Sobin and Wittekind, 1997). The clinical characteristics of the patients are presented in Table 1

**Table 1:** Relation of MRP-1/CD9, KAI1/CD82 and CD151 expression and various prognostic factors in 146 patients with colon cancer

		**MRP-1/CD9**			**KAII/CD82**			**CD151**		
**Clinicopathological characteristics**	**Number of patients**	**Positive**	**Negative**	***P*-value**	**Positive**	**Negative**	***P*-value**	**Positive**	**Negative**	***P*-value**
Age (years)										
⩽60	74	35	39	NS	33	41	NS	35	39	NS
⩾60	72	29	43		30	42		46	26	
										
Sex										
Female	62	29	33	NS	29	33	NS	32	30	NS
Male	84	35	49		34	50		49	35	
										
Tumour status										
T1	17	9	8	NS	11	6	NS	9	8	0.025
T2	20	8	12		7	13		12	8	
T3	83	38	45		38	45		39	44	
T4	26	9	17		7	19		21	5	
										
Nodal status										
N0	71	39	32	0.007	43	28	<0.001	37	34	NS
N1	33	14	19		11	22		15	18	
N2	42	11	31		9	33		29	13	
										
Pathological stage										
I	25	13	12	0.030	15	10	<0.001	15	10	NS
II	46	26	20		28	18		22	24	
III	75	25	50		20	55		44	31	
										
Differentiation										
Well	32	16	16	NS	18	14	NS	14	18	NS
Moderately	101	42	59		40	61		57	44	
Poorly	13	6	7		5	8		10	3	
										
Total	146	64	82		63	83		81	65	

NS=not significant.

. In all, 84 of patients were men and 62 were women. The median age of the patients was 62.8 years, with a range of 35–80 years. The patients could be broken down into 25 with pathological stage I, 46 with stage II and 75 with stage III disease. The mean follow-up period for all patients was 44.3 months, with a range of 6.3–85.9 months.

### Tumour specimens

One-half of each fresh tumour tissue specimen was immediately embedded in optimum cutting temperature compound (Miles, Kankakee, IL, USA), and frozen in liquid nitrogen immediately after surgical resection and maintained at −80°C until use. Frozen sections were cut on a cryostat to a thickness of 6 *μ*m and were stained with haematoxylin and eosin and used for immunohistochemical staining. After the connective tissues were trimmed off, the other-half of the tumour specimen that was then made up of more than 80% cancer cells was used for the reverse transcriptase–polymerase chain reaction (RT–PCR) analysis.

### Immunohistochemical assays

The assays were carried out as described previously (Hashida *et al*, 2002a). Endogenous peroxidases were blocked by incubating with 0.3% H_2_O_2_ in absolute methanol for 30 min. The sections were then incubated with 5% bovine serum albumin for 2 h at room temperature. Subsequently, replicate sections were incubated for 2 h with the anti-MRP-1/CD9 MAb M31-15, the anti-KAI1/CD82 MAb C33 and the anti-CD151 MAb SFA1.2B4, respectively. After washing three times in phosphate-buffered saline (PBS), they were then incubated for 1 h with biotinylated horse anti-mouse IgG (Vector Laboratories Inc., Burlingame, CA, USA). Visualisation of the antibody binding was completed using 3,3′-diaminobenzidine tetrahydrochloride, and the sections were lightly counterstained with Mayer's haematoxylin. Sections incubated with mouse myeloma SP2 supernatant and mouse IgG were used as negative reaction control. Specimens of fibroadenoma of the breast were used as a positive control.

The samples were classified by two pathologists who had no knowledge of patients' clinical status. All sections were scored in semiquantitative fashion according to the method described previously (Huang *et al*, 1998). Briefly, we determined the score by estimating the percentage of cells that stained for MRP-1/CD9, KAI1/CD82 and CD151, and multiplying by the assessment of the intensity of the stain on a 0, 1+, 2+ or 3+ scale (0, no staining; 1+, weak staining; 2+, distinct staining; 3+, very strong staining). The theoretical limits of the scores ranged from 0 (0% of cells staining) to 300 (100% of the cells staining at 3+ intensity). Significant differences were found with respect to the survival rate when a score of 120 was used as a cut off value. Specimens with a score of ⩾120 were classified as positive, and when score was <120, specimens were classified as negative.

### Semiquantitative RT–PCR analysis

Reverse transcriptase–polymerase chain reaction was performed as described previously in order to confirm the results of MRP-1/CD9, KAI1/CD82 and CD151 expression in immunohistochemical studies (Adachi *et al*, 1996). Total cellular RNA was extracted from the frozen tumour tissues by the acid guanidium thiocyanate procedure (Chomczynski and Sacchi, 1987). First-strand complementary DNA (cDNA) synthesis was performed with 5 *μ*g of total RNA using a cDNA synthesis kit (Pharmacia, Piscataway, NJ, USA) following the manufacturer's protocol. We used 1 *μ*l aliquot of the reaction mixture for PCR amplification. We titrated the amount of starting cDNA and determined the number of amplification cycles to obtain reproducible quantitative performance of the RT–PCR assay for *MRP-1/CD9*, *KAI1/CD82* and *CD151*. The generated cDNA was amplified using primers for *MRP-1/CD9* (5′-TGCATC-TGTATCCAGCGCCA-3′ and 5′-CTCAGGGATGTAAGCTGACT-3′), *KAI1/CD82* (5′-AGTCCTCCCTGCTGCTGTGTG-3′ and 5′-TCAGTCAGGGTGGGCAAGAGG-3′) and *CD151* (5′-ATGGGTGAGTTCAACGAGAAG-3′ and 5′-TCAGTAGTGCTCCAGCTTC-AG-3′). The internal control was *β-actin* (5′-GATATCGCCGCG-CTCGTCGTCGAC-3′ and 5′-CAGGAAGGAAGGCTGGAAGAG-TGC-3′). All of the subsequent assays were then performed under conditions that produced amplifications of *MRP-1/CD9*, *KAI1/CD82*, *CD151* and *β-actin* within a linear range. A total of 24 cycles of PCR amplification was performed for *MRP-1/CD9*, *KAI1/CD82* and *β-actin*, and 20 cycles for *CD151* as follows: denaturation at 94^o^C for 40 s, annealing at 60^o^C for 40 s and extension at 72°C for 90 s. The same PCR conditions were used to amplify the *β-actin* DNA. Tubes containing all of the ingredients except templates were included in all runs and served as negative controls. Preparations of the human endothelial cell line ECV304, which is positive for *MRP-1/CD9*, *KAI1/CD82* and *CD151*, were used as a positive control. The amplified PCR products were electrophoresed on a 1% agarose gel containing ethidium bromide, and the bands were visualised under ultraviolet light followed by densitometric analysis. In addition, the resulting PCR products were analysed by sequencing to confirm their identity as described previously (Onuma *et al*, 1999; Rae *et al*, 2000). PCR products were successfully sequenced. Sequence comparisons coincided with the known MRP-1/CD9, KAI1/CD82 and CD151 genes.

**Figure 1:**
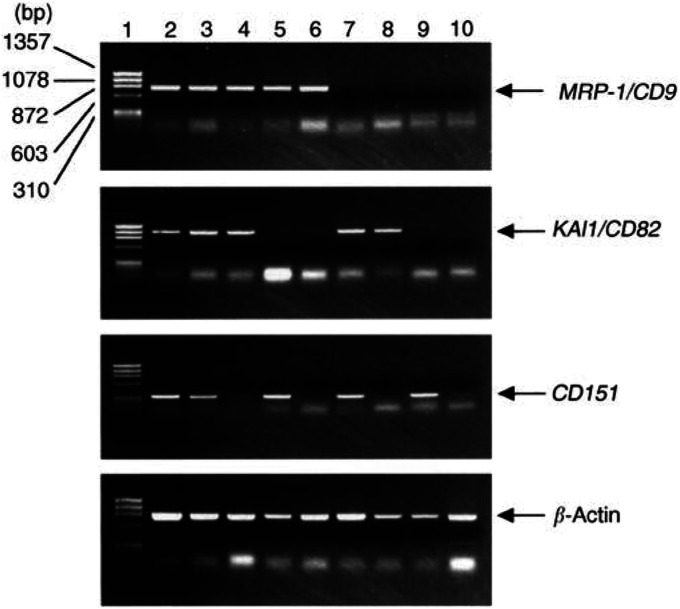
Agarose gel electrophoresis of RT–PCR-amplified *MRP-1/CD9*, *KAII/CD82*, *CD151* and *β*-actin. Lane 1, size marker; lane 2, human endothelial cell line ECV304(positive control); lane 3, colon cancer with *MRP-1/CD9*-, *KAII/CD82*- and *CD151*-positive expression; lane 4, colon cancer with *MPR-1/CD9*- and *KAII/CD82*-positive but *CD151-*negative expression; lane 5, colon cancer with *MRP*-*1/CD9*- and *CD151*-positive but *KAII/CD82*-negative expression; lane 6, colon cancer with *MRP-1/CD9*-positive but *KAII/CD82*- and *CDI5I*-negative expression; lane 7, colon cancer with *KAII/CD82*- and *CD151*-positive but *MRP-1/CD9*-negative expression; lane 8, colon cancer with *KAII/CD82*-positive but *MRP-1/CD9*- and *CD151*-negative expression; lane 9, colon cancer with *CD151*-positive but *MRP-1/CD9*- and *KAII/CD82*-negative expression; lane 10, colon cancer with *MRP-1/CD9*-, *KAII/CD82*- and *CD151*-negative expression.

The densitometric values obtained for *MRP-1/CD9*, *KAI1/CD82* and *CD151* bands in a given tumour tissue sample were divided by the corresponding value of *β-actin* for normalisation, and the ratio was referred to as the gene expression ratio for each gene. The expression ratio of the tumour was then divided by that of the human endothelial cell line ECV304 to obtain the gene conservation rate for *MRP-1/CD9*, *KAI1/CD82* and *CD151*. We arbitrarily set several cutoff values to select the best value. When 0.8 was used as a cutoff value, significant differences were found in survival. We therefore selected 0.8 as the most appropriate cutoff value. When the conservation rate value of a given specimen was ≥0.8, it was considered to indicate positive gene expression. If the value was <0.8, it was considered to indicate negative gene expression (Figure 1

).

### Statistical analysis

The statistical significance of the difference between the incidence of *MRP-1/CD9*, *KAI1/CD82* and *CD151* expression and clinical and pathologic parameters was assessed by the χ^2^ test or Mann–Whitney *U*-test. Overall cancer-specific survival was defined as from the date of surgery to the date of death due to cancer. The Kaplan–Meier method was used to estimate the probability of overall survival as a function of time (Kaplan and Meier, 1958) and was compared using the log-rank test (Mantel, 1966). Multivariate analysis was performed using the Cox regression model (Cox, 1972) to study the effects of different variables on survival, and eight factors (*MRP-1/CD9* status, *KAI1/CD82* status, *CD151* status, sex, age, tumour status, nodal status and histological differentiation) were studied. Scores were assigned to each variable for regression analysis. All *P*-values were based on two-tailed statistical analysis, and a *P*-value of <0.05 was considered to indicate statistical significance.

## RESULTS

### MRP-1/CD9, KAI1/CD82 and CD151 gene expression in colon cancer tissues analysed by RT–PCR

Of the 146 colon cancers studied, 82 carcinomas (43.8%) were evaluated as *MRP-1/CD9*-positive and 64 carcinomas (56.2%) as *MRP-1/CD9*-negative expression. In all, 63 carcinomas (43.2%) were evaluated as *KAI1/CD82*-positive and 83 carcinomas (56.8%) as *KAI1/CD82*-negative expression. A total of 81 carcinomas (55.5%) were evaluated as *CD151*-positive and 65 carcinomas (44.5%) as *CD151*-negative expression (Table 1 and Figure 1).

### Immunohistochemical study of MRP-1/CD9, KAI1/CD82 and CD151 in colon cancer tissues

**Figure 2:**
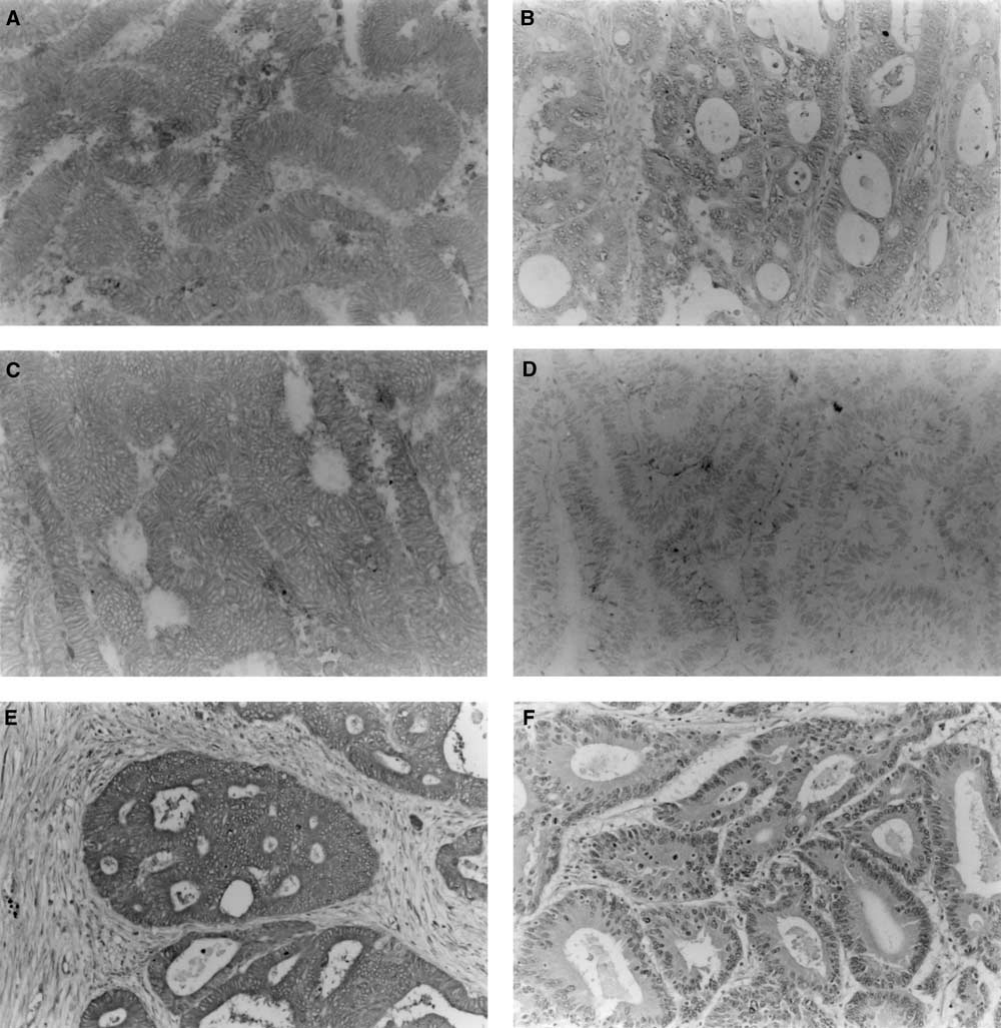
Immunohistochemical staining of human colon cancer tissues using the avidin–biotin–peroxidase complex procedure (original magnification, × 150). (**A**) *MRP-1/CD9-*positive staining of colon cancer; (**B**) *MRP-1/CD9* negative staining of colon cancer; (**C**) *KAII/CD82*-positive staining of colon cancer; (**D**) *KAI1/CD82*-negative staining of colon cancer; (**E**) *CD151*-positive staining of colon cancer; (**F**) *CD151*-negative staining of colon cancer.

Of the 146 colon cancers studied using the immunohistochemical method, 69 (49.1%) were classified as MRP-1/CD9 positive (Figure 2A

), and the immunostaining was intense and uniform on the cell surface membrane in these specimens. There were 77 specimens (50.9%) with negative MRP-1/CD9 expression (Figure 2B). The immunohistochemical results agreed well with those from the RT–PCR assays, and 92.5% of the specimens coincided exactly.

Turning to the KAI1/CD82, there were 67 specimens (45.9%) with positive KAI1/CD82 expression and 79 specimens (54.1%) with negative KAI1/CD82 expression (Figure 2C, D). These results agreed well with those from the RT–PCR assays, and 93.4% of the specimens coincided exactly.

Furthermore, there were 78 specimens (53.4%) with positive CD151 expression and 68 specimens (46.6%) with negative CD151 expression (Figure 2E, F). These results agreed with those from the RT–PCR assays, and 88.9% of the specimens coincided exactly.

### Relation between MRP-1/CD9, KAI1/CD82 and CD151 expression and various prognostic factors

The relation between *MRP-1/CD9* gene expression and various prognostic factors is shown in Table 1. The relation was found between *MRP-1/CD9* gene expression and nodal status (*P*=0.007) and pathological stage (*P*=0.030). In all, 32 (45.1%) patients with N0 stage had negative gene expression compared with 57.6% of N1 stage patients and 75.6% of N2 stage patients. In addition, the percentage of patients whose tumours had *MRP-1/CD9*-negative gene expression increased from 48.0% of those with stage I to 66.7% of those with stage III.

In contrast, *KAI1/CD82* gene expression was associated with lymph node status (*P<*0.001) as well as pathological stage (*P<*0.001). A total of 28 (39.4%) patients with N0 stage had negative gene expression compared with 66.7% of N1 stage patients and 78.6% of N2 stage patients. With respect to pathological stage, the percentage of patients whose tumours had *KAI1/CD82*-negative gene expression increased from 40.0% of those with stage I to 73.3% of those with stage III.

The relation between *CD151* gene expression and various prognostic factors is shown in Table 1. There was a statistically significant relation between gene expression and tumour status (*P*=0.025). Eight patients (47.1%) with T1 stage had negative gene expression compared with 19.2% of T4 stage patients.

### Relation between MRP-1/CD9 expression and 3-year, disease-free and overall survival of colon cancer patients

Among all 146 patients, the 3-year survival rate of patients with *MRP-1/CD9*-positive tumours was significantly higher than that of patients with *MRP-1/CD9*-negative tumours (98.1 *vs* 76.0%, *P<*0.001; Table 2

**Table 2:** Survival rate of 146 patients with colon cancer according to clinicopathological characteristics and MRP-1/CD9

	**3-year survival rate (%)**			**Disease-free** **survival rate (%)**			**Overall survival rate (%)**		
**Clinicopathological characteristics**	**Positive**	**Negative**	***P*-value**	**Positive**	**Negative**	***P*-value**	**Positive**	**Negative**	***P*-value**
Age (years)									
⩽60	96.4	87.2	0.185	78.1	41.3	0.032	88.0	56.3	0.056
⩾60	100.0	65.8	0.004	67.5	25.3	0.051	92.3	47.5	0.002
									
Sex									
Female	100.0	83.7	0.049	77.7	44.1	0.057	89.5	63.9	0.044
Male	96.6	71.8	0.009	71.6	40.8	0.057	90.9	41.3	0.002
									
Tumour status									
T1	100.0	100.0	>0.999	100.0	50.0	0.400	100.0	50.0	0.400
T2	100.0	66.7	0.192	77.2	33.3	0.118	80.0	33.3	0.260
T3	100.0	94.4	0.499	75.0	23.3	0.174	95.2	72.4	0.042
T4	83.3	29.4	0.041	50.1	12.3	0.170	55.6	15.7	0.047
									
Nodal status									
N0	100.0	100.0	>0.999	86.6	32.5	0.160	95.2	73.8	0.628
N1	100.0	100.0	>0.999	87.5	64.8	0.328	90.0	77.1	0.458
N2	88.9	35.3	0.012	68.6	10.2	0.045	74.1	12.1	0.003
									
Pathological stage									
I	100.0	100.0	>0.999	72.2	23.7	0.789	100.0	75.0	0.400
II	100.0	100.0	>0.999	85.2	54.4	0.236	91.7	75.0	0.690
III	95.2	60.7	0.006	79.0	34.7	0.013	82.9	37.2	0.002
									
Differentiation									
Well	100.0	86.7	>0.999	83.3	28.4	0.469	100.0	34.2	>0.999
Moderately	97.1	78.7	0.019	80.1	27.8	0.074	85.0	64.5	0.049
Poorly	100.0	20.0	0.015	100.0	0.0	0.008	100.0	0.0	0.008
									
Total	98.1	76.0	<0.001	74.8	43.5	0.004	89.8	50.8	<0.001

). In addition, the disease-free survival rate of patients with *MRP-1/CD9*-positive tumours was significantly higher than that of patients with *MRP-1/CD9*-negative tumours (74.8 *vs* 43.5%, *P*=0.004; Table 2). Similarly, the overall survival rate for patients with positive tumours was significantly better than that of individuals whose tumours had negative *MRP-1/CD9* expression (89.8 *vs* 50.8%, *P<*0.001; Table 2 and Figure 3

**Figure 3:**
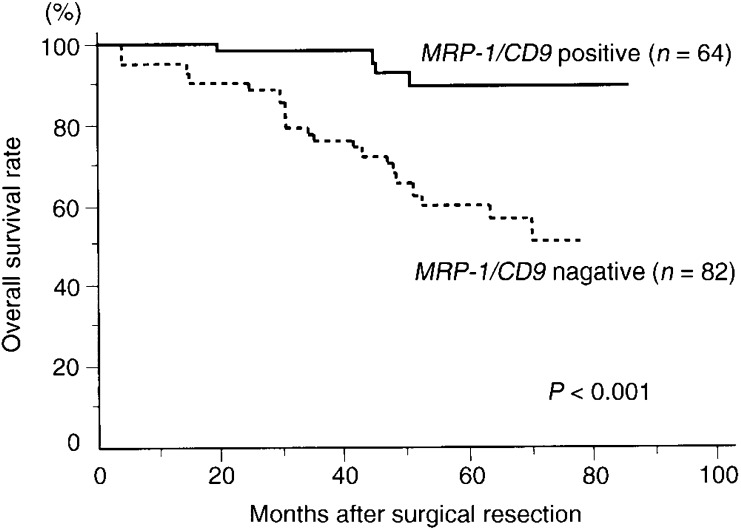
Overall survival of 146 colon cancer patients according to their tumour *MRP-1/CD9* gene status.

). In particular, *MRP-1/CD9* was an effective indicator for patients with advanced disease (N2 status and stage III).

### Relation between KAI1/CD82 expression and 3-year, disease-free and overall survival of colon cancer patients

Among the 146 patients, the 3-year survival rate of patients with *KAI1/CD82*-positive tumours was significantly higher than that of patients with *KAI1/CD82*-negative tumours (98.2 *vs* 75.7%, *P<*0.001; Table 3

**Table 3:** Survival rate of 146 patients with colon cancer according to clinicopathological characteristics and KAI1/CD82

	**3-year survival rate (%)**			**Disease-free survival rate (%)**			**Overall survival rate (%)**		
**Clinicopathological characteristics**	**Positive**	**Negative**	***P*-value**	**Positive**	**Negative**	***P*-value**	**Positive**	**Negative**	***P*-value**
Age (years)									
⩽60	96.6	87.1	0.176	82.1	47.2	0.080	86.8	62.8	0.118
⩾60	100.0	63.7	0.001	50.8	36.7	0.020	78.7	51.5	0.006
									
Sex									
Female	100.0	84.8	0.112	100.0	33.3	0.021	100.0	60.6	0.013
Male	96.7	69.8	0.006	57.7	42.3	0.232	73.9	44.5	0.051
									
Tumour status									
T1	100.0	100.0	>0.999	100.0	0.0	0.250	100.0	0.0	0.200
T2	100.0	70.5	0.209	100.0	0.0	0.036	100.0	0.0	0.048
T3	100.0	94.2	0.495	75.0	66.3	0.413	81.2	83.7	0.863
T4	80.0	33.8	0.115	35.6	20.3	0.480	40.0	22.1	0.190
									
Nodal status									
N0	100.0	100.0	>0.999	75.0	22.7	>0.001	100.0	42.6	0.048
N1	100.0	100.0	>0.999	71.4	65.9	0.823	75.0	87.5	0.539
N2	87.5	38.1	0.028	27.8	22.2	0.610	43.8	24.2	0.082
									
Pathological stage									
I	100.0	100.0	>0.999	66.7	0.0	<0.001	100.0	66.7	0.300
II	100.0	100.0	>0.999	100.0	40.9	0.236	100.0	45.8	0.400
III	94.1	64.2	0.025	53.1	45.6	0.556	61.0	49.1	0.173
									
Differentiation									
Well	100.0	84.6	0.192	75.0	18.8	0.035	83.3	30.8	0.138
Moderately	97.1	78.7	0.020	55.5	46.5	0.126	87.4	62.4	0.021
Poorly	100.0	33.3	0.061	75.0	25.6	0.090	75.0	33.3	0.103
									
Total	98.2	75.7	<0.001	77.1	42.3	<0.001	84.8	54.9	0.002

). In addition, the disease-free survival rate of patients with *KAI1/CD82*-positive tumours was significantly higher than that of patients with *KAI1/CD82*-negative tumours (77.1 *vs* 42.3%, *P*=0.004; Table 3). Moreover, the overall survival rate for patients with positive tumours was significantly better than that of individuals whose tumours had negative *KAI1/CD82* expression (84.8 *vs* 54.9%, *P*=0.002; Table 3 and Figure 4

**Figure 4:**
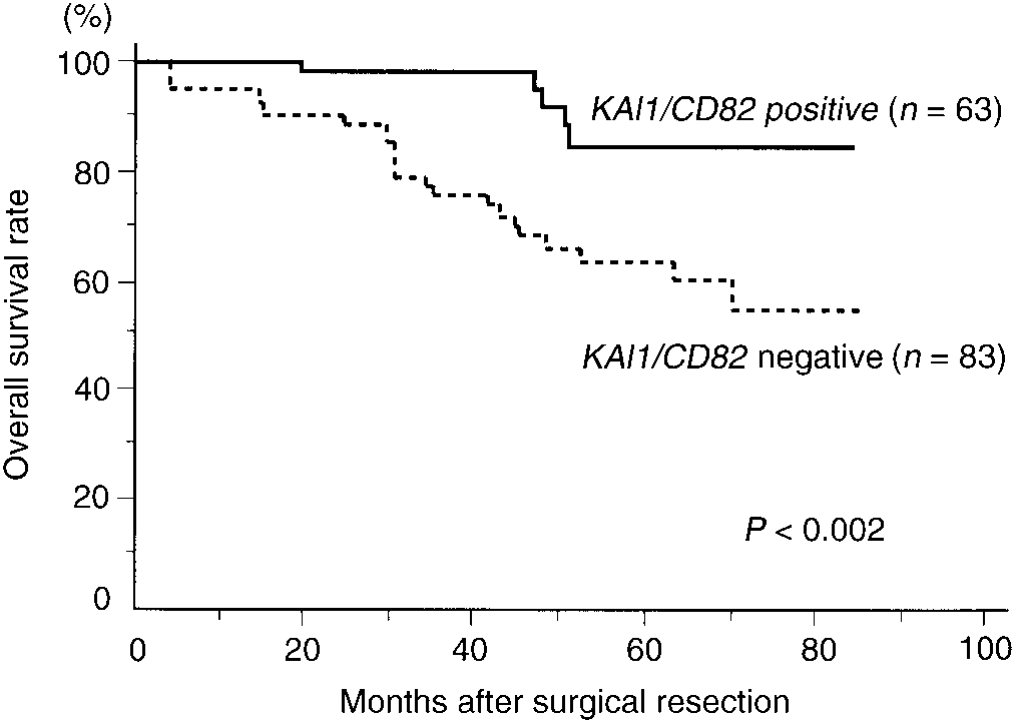
Overall survival of 146 colon cancer patients according to their tumour *KAII/CD82* gene status.

).

### Relation between CD151 expression and 3-year, disease-free and overall survival of colon cancer patients

Among all 146 patients, the 3-year survival rate of patients with *CD151*-positive tumours was significantly lower than that of patients with *CD151*-negative tumours (78.5 *vs* 94.3%, *P*=0.012; Table 4

**Table 4:** Survival rate of 146 patients with colon cancer according to clinicopathological characteristics and CD151

	**3-year survival rate (%)**			**Disease-free survival rate (%)**			**Overall survival rate (%)**		
**Clinicopathological characteristics**	**Positive**	**Negative**	***P*-value**	**Positive**	**Negative**	***P*-value**	**Positive**	**Negative**	***P*-value**
Age (years)									
⩽60	87.5	94.3	0.298	57.8	66.1	0.281	67.9	75.4	0.213
⩾60	70.7	94.4	0.030	49.3	62.4	0.118	58.4	72.6	0.087
									
Sex									
Female	92.3	91.5	0.900	60.7	60.0	0.466	75.2	79.3	0.887
Male	69.3	96.7	0.005	49.4	61.2	0.065	51.6	72.6	0.010
									
Tumour status									
T1	100.0	100.0	>0.999	50.0	100.0	0.400	50.0	100.0	0.400
T2	80.0	83.3	0.853	62.3	40.0	0.653	66.7	40.0	0.805
T3	96.4	97.4	0.914	60.2	56.6	0.159	83.2	88.3	0.887
T4	33.8	80.0	0.115	16.9	55.3	0.451	16.9	53.3	0.159
									
Nodal status									
N0	100.0	100.0	>0.999	84.8	78.5	0.447	88.1	80.0	0.418
N1	100.0	100.0	>0.999	56.1	62.3	0.797	70.0	92.9	0.150
N2	42.3	72.5	0.111	30.4	0.0	0.371	32.9	0.0	0.726
									
Pathological stage									
I	100.0	100.0	>0.999	85.7	100.0	>0.999	85.7	100.0	>0.999
II	100.0	100.0	>0.999	80.1	51.1	0.071	90.0	75.0	0.589
III	60.4	88.9	0.010	40.7	40.7	0.147	42.6	66.4	0.033
									
Differentiation									
Well	85.7	100.0	0.483	40.9	42.9	0.266	42.9	45.0	0.158
Moderately	77.0	97.5	0.008	50.3	64.3	0.033	58.9	89.0	0.006
Poorly	75.0	33.3	0.188	64.3	0.0	0.123	75.0	0.0	0.034
									
Total	78.5	94.3	0.012	53.6	61.2	0.048	61.2	74.9	0.022

). In addition, the disease-free survival rate of patients with *CD151*-positive tumours was significantly lower than that of patients with *CD151*-negative tumours (53.6 *vs* 61.2%, *P*=0.048; Table 4). Moreover, the overall survival rate for patients with negative tumours was significantly better than that of individuals whose tumours had positive *CD151* expression (61.2 *vs* 74.9%, *P*=0.022; Table 4 and Figure 5

**Figure 5:**
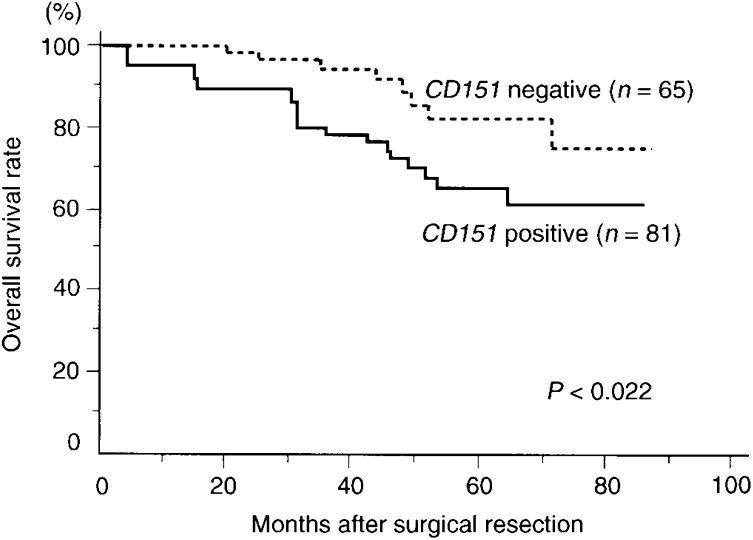
Overall survival of 146 colon cancer patients according to their tumour *CD151* gene status.

).

### Prognostic value of MRP-1/CD9, KAI1/CD82 and CD151 expression

The Cox regression model was used to evaluate disease-free and overall survival as shown in Table 5

**Table 5:** Multivariate regression analysis in predicting the disease-free and overall survival of 146 patients with colon cancer

		**Disease-free survival**		**Overall survival**	
**Variables**	**Assigned score**	**Hazard ratio (95% CI)**	***P*-value**	**Hazard ratio (95% CI)**	***P*-value**
*MRP-11CD9*					
Positive	0	3.147(1.240–7.940)	0.015	5.511(1.584–19.158)	0.007
Negative	1				
					
*KAI1/CD82*					
Positive	0	0.949 (0.388–2.321)	0.909	1.107 (0.364–3.369)	0.858
Negative	1				
*CD151*					
Negative	0	0.529 (0.247–1.123)	0.097	0.603 (0.028–1.597)	0.309
Positive	1				
					
Age (years)					
⩽60	0	1.491 (0.748–2.974)	0.257	1.258(0.569–2.782)	0.571
⩾60	1				
					
Sex					
Female	0	1.374 (0.699–2.702)	0.357	1.838 (0.786–4.300)	0.160
Male	1				
					
Tumour status					
T1	1	1.561 (0.942–2.585)	0.084	2.066 (1.101–3.874)	0.024
T2	2				
T3	3				
T4	4				
					
Nodal status					
N0	0	2.812 (1.736–4.556)	<0.001	3.797 (1.981–7.277)	<0.001
N1	1				
N2	2				
					
Differentiation					
Well	0	0.811 (0.422–1.559)	0.530	0.815(0.384–1.728)	0.593
Moderately	1				
Poorly	2				

Abbreviation: CI=confidence interval.

. Two variables, nodal status (*P<*0.001) and *MRP-1/CD9* status (*P*=0.015), were significant factors in predicting the disease-free survival of colon cancer patients. Moreover, three variables, nodal status (*P<*0.001), tumour status (*P*=0.029) and *MRP-1/CD9* status (*P*=0.015), were significant factors in predicting the overall survival of colon cancer patients.

## DISCUSSION

Colon cancer has become one of the most common causes of cancer death in Japan (Health and Welfare Statistics Association, 1998). Endoscopic treatments for early stage of colon cancer have been improved, but no established treatment is available for patients in the advanced stage. Up until now, the prognosis of patients with colon cancer has depended on the clinicopathological staging. However, recently, molecular assessment has been incorporated into treatment of patients with colon cancer (Boland *et al*, 2000). It is well known that the accumulation of genetic alterations facilitates the progression of tumours. The famous model of colorectal tumorigenesis shows that three tumour suppressor genes, *p53*, *APC* and *DCC*, and the dominant oncogene K*-ras* play key roles in the progression of colon cancer (Feason and Vogelstein, 1990). In addition, many other prognostic factors have been examined for colon cancer. However, little is understood about the genetic alternations and cellular mechanisms that are responsible for the final steps in tumour metastasis (Haier *et al*, 2000). An enhanced understanding of the molecular genetic events that occur during progression in colon cancer could lead to new therapeutic modalities and improvement in survival rates.

In the present study, we investigated the gene expressions of the three TM4SF members, *MRP-1/CD9*, *KAI1/CD82* and *CD151*. This TM4SF is made up of approximately 23 members. These proteins are variously expressed in leucocytes and a variety of non-haematopoietic tissues. MRP-1/CD9 is a glycoprotein widely expressed not only in haematopoietic tissues, platelets, early B cells, activated T cells and granulocytes, but also in nonhaematopoietic tissues (Wright and Tomlinson, 1994). CD9 null female displays a severe reduction in fertility and CD9 appears to be essential for sperm–egg fusion, a process involving the CD9-associated integrin *α*6*β*1 (Le Naour *et al*, 2000). TM4SF proteins act as links between extracellular integrins and intracellular signalling molecules, such as phosphatidylinositol 4-kinase (Yauch *et al*, 1998) and TM4SF proteins appear to regulate cell motility through this link (Hemler, 1996; Berditchevski and Odintsova, 1999). Several studies suggest that TM4SF plays an important role in the regulation of cell development, activation, proliferation and adhesion (Wright and Tomlinson, 1994; Hemler *et al*, 1996; Hashida *et al*, 2002b). Interestingly, several TM4SF member proteins have been associated with the metastatic phenotype, but these associations have been, for the most part, negative. Previously, we showed that reductions of *MRP-1/CD9* and *KAI1/CD82* were predictive factors for a poor prognosis in patients with various kinds of cancers. As part of our evaluation of members of TM4SF as possible prognostic predictors, we further extended our study to the expression of *CD151* and performed a retrospective study on the expression of *CD151*, *MRP-1/CD9* and *KAI1/CD82* in colon cancer. CD151 was reported to be a metastasis-associated antigen that appeared to contribute positively to the metastatic phenotype in contrast to the MRP-1/CD9, KAI1/CD82 and ME491/CD63 (Testa *et al*, 1999). In addition, it was reported that CD151 may not affect tumour cell proliferation but could be involved in an early step in the formation of secondary metastatic lesions. The ability of CD151 to mediate tumour cell migration may provide a possible mechanism for the role of this protein in effecting metastatic dissemination. Moreover, it was reported that the CD151 molecule enhances cell motility, invasion and metastasis of cancer cells and that focal adhesion kinase is needed for these events through CD151 (Kohno *et al*, 2002). We have hypothesised that those patients with a CD151-positive tumour are in a more advanced stage of the disease and have a much poorer prognosis. In this study, the *CD151* mRNA levels and protein expression were associated with tumour status. Moreover, our present study showed that there was a significant difference between the overall survival rate for colon cancer patients with low CD151 expression and that of patients with CD151-positive tumours. These findings suggest that CD151 may play an important role in the progression in colon cancer cells, and that its expression may have an effect on the characteristics of colon cancers. Further basic studies using an experimental metastasis model will be necessary.

On the other hand, KAI1/CD82 expression was demonstrated to be consistently downregulated during the progression of human colon cancer, as well as breast cancer (Huang *et al*, 1998), pancreatic cancer (Sho *et al*, 1998) and nonsmall cell lung cancer (Adachi *et al*, 1996). Furthermore, we have shown that the survival rate for colon cancer patients with negative KAI1/CD82 expression was strikingly lower than that of patients with KAI1/CD82-positive tumours. Similarly, the survival rate of patients with MRP-1/CD9-negative tumours was significantly lower than that of patients with MRP-1/CD9-positive tumours. The malignancy-suppressing effect of CD82 or CD9 may be based partially on cell motility inhibition and apoptosis induction promoted by concurrent GM3 synthesis and *N*-glycosylation (Ono *et al*, 1999). Although the precise mechanism remains unknown, the levels of the *MRP-1/CD9* or *KAI1/CD82* gene may be diminishing due to promoter abnormality or aberrant glycosylation may have occurred in the first hydro-philic region and normal MRP-1/CD9 or KAI1/CD82 function could be lost. Hence, GM3 and other lipids may contribute to the formation of tetraspanin/tetraspanin complexes. Our present study suggests that cancer progression may lead to collapse of tetras-panin/tetraspanin complexes as well as the collapse of tetraspanin/integrin complexes. In addition, a link between a tetraspanin and Rho GTPase cascade may explain why members of TM4SF are involved in cell activation, adhesion, growth and metastasis (Delaguillaumie *et al*, 2002). Although the precise biological functions of these proteins remain unknown, the downregulation of *KAI1/CD82* and *MRP-1/CD9* genes during the progression of human cancer is highly associated with a poor prognosis.

The classification of colon cancers according to MRP-1/CD9, KAI1/CD82 and CD151 expression might be useful in identifying patients for whom intensive adjuvant therapy is warranted. It is conceivable that testing tumours for TM4SF expression, in combination with other molecular and biochemical assays, may improve the prognostic evaluation of colon cancer patients, and enhance the clinician's ability to prospectively identify patients who will have early disease recurrence and who require adjuvant chemotherapy.
